# Response mechanisms of bacterial communities and nitrogen cycle functional genes in millet rhizosphere soil to chromium stress

**DOI:** 10.3389/fmicb.2023.1116535

**Published:** 2023-02-22

**Authors:** Xue Bai, Yvjing Li, Xiuqing Jing, Xiaodong Zhao, Pengyu Zhao

**Affiliations:** Department of Biology, Taiyuan Normal University, Taiyuan, China

**Keywords:** heavy metal stress, microbial community, high-throughput sequencing, qPCR, PLS-EM

## Abstract

**Introduction:**

A growing amount of heavy metal contamination in soil disturbs the ecosystem’s equilibrium, in which microbial populations play a key role in the nutrient cycle of soils. However, given the different sensitivity of microbial communities to different spatial and temporal scales, microbial community structure and function also have varied response mechanisms to different heavy metal contaminated habitats.

**Methods:**

In this study, samples were taken prior to Cr stress (CK) and 6 h and 6 days after Cr stress (Cr_6h, Cr_6d) in laboratory experiments. High-throughput sequencing revealed trends in the structure and diversity of the bacterial communities, and real-time fluorescence quantitative polymerase chain reaction (qPCR) was used to analyze trends in nitrogen cycle functional genes (*AOA-amoA*, *AOB-amoA*, *narG*, *nirK*, and *nifH*).

**Results:**

The findings showed that (1) the composition structure of the soil bacterial community changed considerably in Cr–stressed soils; α-diversity showed significant phase transition characteristic from stress to stability (*p* < 0.05). (2) With an overall rising tendency, the abundance of the nitrogen cycle functional genes (*AOA-amoA* and *AOB-amoA*) decreased considerably before increasing, and α-diversity dramatically declined (*p* < 0.05). (3) The redundancy analysis (RDA) and permutational multivariate analysis of variance (PERMANOVA) tests results showed that the soil physicochemical parameters were significantly correlated with the nitrogen cycle functional genes (*r*: 0.4195, *p* < 0.01). Mantel analysis showed that available nitrogen (N), available potassium (K), and available phosphorus (P) were significantly correlated with *nifH* (*p* = 0.006, 0.008, 0.004), and pH was highly significantly correlated with *nifH* (*p* = 0.026). The PLS-ME (partial least squares path model) model further demonstrated a significant direct effect of the soil physicochemical parameters on the nitrogen cycling functional genes.

**Discussion:**

As a result, the composition and diversity of the bacterial community and the nitrogen cycle functional genes in Cr–stressed agricultural soils changed considerably. However, the influence of the soil physicochemical parameters on the functional genes involved in the nitrogen cycle was greater than that of the bacterial community. and Cr stress affects the N cycling process in soil mainly by affecting nitrification. This research has significant practical ramifications for understanding the mechanisms of microbial community homeostasis maintenance, nitrogen cycle response mechanisms, and soil remediation in heavy metal–contaminated agricultural soils.

## 1. Introduction

Chromium (Cr) has remained one of the most prevalent heavy metal pollutants in groundwater and soil since the industrialization of society in the middle and late 20th century as a result of mining, metallurgy, and tanning (Chen et al., 2014). According to studies, China has a total exceedance rate of 16.1% for heavy metal contamination in agricultural soils, and the amount of arable land that has been contaminated by Cr has surpassed 20 million hm^2^ (square hectometer) (Duan et al., 2016). As the main heavy metal pollutant in China’s soil, Cr can enter the organism through the cell plasma membrane to change the genetic material or disrupt the expression of metal proteins due to its high mobility, toxicity, persistence, and solubility (Mathivanan et al., 2021), posing a major threat to soil microbes, plant growth, and food safety. As a result, heavy metal contamination has emerged as a challenging aspect of environmental management and has sparked global concern.

Although microbial communities in soils only make up a small portion of organic matter, they play an important role in energy flow, nutrient cycling, material transformation, and pollutant degradation and are highly sensitive to heavy metals (Xie et al., 2016). Therefore, the accumulation of heavy metals in soils impacts the structure and function of soil ecosystems, and their structural and functional alterations are reliable indicators of soil quality and ecological functions (Wu et al., 2017). Heavy metal pollution, such as that caused by lead (Pb), cadmium (Cd), copper (Cu), or complex heavy metals, greatly affects the abundance, composition, and diversity of microbial communities in soil, and the degree of the impact is directly correlated with the type of heavy metals, heavy metal concentration microbial communities can improve plant tolerance by reducing the effectiveness of heavy metals in soil (Zhang et al., 2016; Huang et al., 2021; Mao et al., 2022). Additionally, soil physicochemical properties have a close relationship with soil microbial communities, which act as key nutrient regulators. The abundance and diversity of the microbial community are strongly linked to soil organic carbon (SOC), pH, available nitrogen (N) and available phosphorus (P), indicating that heavy metals and soil physicochemical properties have a synergistic effect on the structure and function of the microbial community (Deng et al., 2015; Kasemodel et al., 2019; Hu et al., 2021). As a result, research on the methods by which soil microbial communities respond to heavy metal stress is a hot topic right now, but there is presently a dearth of data on the microbial communities in soils that are stressed by Cr.

One of the components necessary for microbial growth is nitrogen (N), which is also critical for soil nitrogen supply, crop growth, and food security (Robertson and Vitousek, 2009). Soil nitrogen turnover is a crucial aspect of agroecosystems. In addition, the nitrogen cycle is a network of redox processes of nitrogen compounds that are catalyzed jointly by plants and bacteria, fungus, and archaea (Devrim et al., 2017). Soil microorganisms are usually involved in the main processes of the nitrogen cycle such as nitrogen fixation, ammonification, nitrification, and denitrification. Moreover, soil microorganisms constitute the primary driving force in the nitrogen cycle process, which can directly influence the process of nitrogen transformation and supply (Van der Heijden et al., 2008). Among soil microorganisms, the nitrogen cycle mediated by nitrogen-fixing bacteria and nitrifying bacteria plays an important role in ecosystem function (Kuypers et al., 2018). Through the expression of functional genes such as encoding key enzymes, nitrogen cycle functional microorganisms in soil can regulate the soil nitrogen cycle, and the abundance of their genes is frequently used to estimate the number of functional microorganisms and population fluctuations related to the soil nitrogen cycle (Li X. M. et al., 2020). The investigation of the ecological processes of the nitrogen cycle and associated functional microorganisms or functional gene clusters is one of the hottest areas of current microbial ecology research.

Numerous studies have examined the ecological processes of nitrogen cycling in various habitats by examining the expression of genes such as *nifH* (nitrogen-fixing enzyme), *amoA* (ammonia monooxygenase), and *narG* (nitrate reductase) during nitrogen fixation, nitrification, and denitrification (Ouyang et al., 2018; Li P. P. et al., 2019, 2020). For example, high levels of heavy metal contamination may lead to greater functional diversity and abundance of resistance genes to strengthen microbial community resistance (Jie et al., 2016); *narG* gene abundance in biochar-remediated heavy metal-contaminated soils was most sensitive to changes in soil samples’ physicochemical properties (Li M. Y. et al., 2019); the abundance of bacterial functional genes associated with the carbon and nitrogen cycles increased in Cd-contaminated soils with soil amendments applied by GeoChip; and in eutrophic soils with urea addition under Cd stress conditions, Cd altered microorganisms’ C/N metabolism from catabolic to anabolic dominance. However, there is little research on the functional microbial communities associated with nitrogen cycling in Cr-contaminated soils (Xu et al., 2021). The nitrogen transformation process of microbial communities induced by heavy metal pollution is controlled by a variety of soil physicochemical parameters and complicated interactions between organisms (Giannopoulos et al., 2020; Rijk and Ekblad, 2020), and the sensitivity of nitrogen cycling-related microbial communities to different geographical and temporal scales is diverse, so their response processes are difficult to predict.

Because ammonia-oxidizing and denitrifying microorganisms serve as model organisms, both functional gene abundances are frequently employed to predict the potential rates of total soil nitrification and denitrification (Wang et al., 2022); additionally, the ammonia-oxidizing process consists only of a small number of phylogenetically aggregated ammonia-oxidizing archaea (AOA) and ammonia-oxidizing bacteria (AOB), which are better suited for investigating their relationship with the ecosystem (Khanal and Lee, 2020). Therefore, in this study, we collected rhizosphere soil samples of cereals under Cr stress and used high-throughput sequencing analysis and the real-time fluorescence quantitative PCR (q-PCR) technique to analyze the microbial community and the abundance of functional nitrogen cycle genes (*nifH*, *AOA-amoA*, *AOB-amoA*, *narG*, *nirK* [nitrate reductase]) in soil. In order to analyze the impacts of Cr stress on the nitrogen cycle in agricultural soils holistically, it is helpful to study the mechanisms by which functional microbial communities in the rhizosphere soils of cereals respond to time series of Cr stress. This research provides a theoretical framework for anticipating how microorganisms will respond, adapt, and feedback to heavy metal contamination in the environment, which is crucial for attaining sustainable agriculture and soil management.

## 2. Materials and methods

### 2.1. Experimental design and sample collection

The seeds used for planting were of the high-quality, high-yielding cereal ‘Jingu 21,’ the most recent variety grown by the Shanxi Academy of Agricultural Science and the Industrial Crop Institute; the soil used for planting was grass charcoal substrate soil (for seedlings). And according to previous research (Sharma et al., 2020), the reagent used in the experiments was 1 mmol∙L^−1^ of Cr^6+^ (K_2_Cr_2_O_7_) to ensure that high concentrations of Cr were toxic to cereal crops.

In this study’s greenhouse pot experiment, the test soil was cleaned of stones and other contaminants before being left aside and air-drying for 3 days in the laboratory. Selected high-quality seeds were cleaned with tap water, shielded from light, and immersed in water for approximately 48 h to promote germination. For the planting process, 18 pots (15 cm × 20 cm) were used, and the soil moisture was regulated to 60% of the water-holding capacity required for crop growth. Each pot included 1,300 g of soil and 50–60 seeds that were uniformly dispersed, and the top layer was covered with soil to protect the seeds from light and promote germination and was wrapped with plastic wrap to prevent contamination by airborne bacteria. The pots were then placed in a growth chamber at a constant temperature of 23°C with 16–8 h of light and dark per day. After 15–20 days of incubation, each pot received 300 ml of 1 mmol·L^−1^ Cr^6+^ (The Cr^6+^ solution was added to the trays and applied every other day at a fixed time. And distilled water was sprayed on the soil surface daily to ensure soil moisture, no fertilizer was applied to the soil during the experiment).

Three groups made up the experiment: the group used before treatment with Cr solution are the control (CK, equal amount of distilled water was applied to the control group), Cr stress treatment for 6 h (Cr_6h), and Cr stress treatment for 6 days (Cr_6d) (Leng et al., 2020). The setting of the sampling time was done because the microbial community in the soil will respond to the heavy metal stress to a certain extent when Cr stress is applied for approximately 6 h. Due to its inherent resistance and resilience, the microbial community eventually displayed a stable state after around 6 days of the Cr stress treatment. No follow-up analysis was done since heavy metal pollution is a long-term problem. Each experimental group had six biological replicates, and the soil in each pot was randomly selected as the sampling target for rhizosphere soil, for a total of 18 samples. The sampling process was carried out by gently grating the soil with a small spade to expose all the roots of the plant as much as possible and to ensure the integrity of the root soil. The cereal seedlings were removed as a whole and the bulk soils of the root system were shaken off, and about 1 g of soil was collected from each plant. The collected soil was stored in sealed sterile bags and marked properly. The sterile bags were placed in a biological sample sampling box for low temperature storage (in advance in ice packs or dry ice). All samples were then stored in a −80°C refrigerator until use; some were used for DNA extraction and some for determination of soil physical and chemical indicators.

### 2.2. Determination of soil physicochemical parameters and plant physiological indicators

The physical and chemical properties of soil were measured with a JXBS-3001-SCPT-SC (Weihai, Shandong Province, China), which is a high-precision environmental sensor that determines available nitrogen (N), available phosphorus (P), available potassium (K), electrical conductivity (EC) and pH in soil.

A Beijing Zhongke Weihe chlorophyll meter (TYS-3 N, Haidian District, Beijing, China), a pressure-head sensor that emits red and infrared light with peak wavelengths of 650 mm and 940 mm, respectively, was used to measure the chlorophyll content and nitrogen content of the leaves of living seedlings. The samples were measured for stem length, root length, and fresh weight; dried and heated at 105°C for 30 min, then dried at 60°C until a constant weight to calculate their dry weight, after which they were photographed to record seedling growth.

### 2.3. Soil DNA extraction, quantitative polymerase chain reaction, and high-throughput sequencing analysis

A TIANamp Bacteria DNA kit (Tiangen Biochemical Technology Co., Beijing, China) was used to extract and purify genomic DNA from the samples, which totaled 1 g of soil. Agarose gel electrophoresis was used to assess the extracted DNA, and a enzyme-labeled instrument (Epoch, Xinghui Biotechnology Co., Ltd., Tianjin, China) was used to gauge the extracts’ purity and quantity.

Genomic DNA was amplified by polymerase chain reaction (PCR). The following components made up the amplification system (50 μl): Taq DNA polymerase (5 U∙μL-1) 1.0 μl, 10 × PCR buffer 10.0 μl, dNTP solution 8.0 μl, template DNA 10.0 μl and primers (50 μmol∙L-1). PCR reaction conditions: pre-denaturation at 98°C for 1 min, followed by 98°C for 10 s, 50°C for 30 s, and 72°C for 30 s, for a total of 30 cycles; with a final extension at 72°C for 5 min (Zeng and An, 2021).

The PCR products were then delivered to Shanghai Meiji Biotechnology Engineering Co., Ltd. for Illumina MiSeq PE300 platform high-throughput sequencing analysis. The bacterial community in soil was sequenced using the sequence amplification region 338F_806R (338F: ACTCCTACGGGAGGCAGCAG; 806R: GGACTACHVGGGTWTCTAAT) (Caporaso et al., 2011), and the raw data from the sequencing platform were homogenized. The Flash (1.2.11^1^) and Fastp (0.19.6^2^) software programs were used to perform double-end sequence splicing, filter the reads’ tails for bases with quality values below 20, and eliminate reads that contained N bases to produce high-quality sequences. The overlap between PE readings was used to guide the splicing of the sequence. Uparse (7.0.1090^1^) software was used to cluster non-repeated sequences (excluding single sequences) into OTUs based on 97% similarity, and chimeras were eliminated during the process to generate representative sequences of OTUs (operational classification units). Taxonomic analysis of representative sequences of OTUs with a 97% similarity level was carried out using the RDP classifier (2.11^2^) Bayesian technique. Based on OTU abundance data, a annotation analysis was carried out to determine the makeup of the bacterial community in soil samples. Qiime (1.9.1^3^) software was used to create a table of the taxonomic abundance and the calculation of the *β*-diversity distance. Mothur (1.30.2^4^) software was used to conduct the *α*-diversity study.

### 2.4. Real-time fluorescence quantitative PCR

The target segments of five nitrogen cycle functional genes, i.e., nitrogen-fixing enzyme (*nifH*), bacterial ammonia monooxygenase (*AOB-amoA*), archaeal ammonia monooxygenase (*AOA-amoA*), nitrate reductase (*narG*), and nitrite reductase (*nirK*), were quantified by qPCR. In Table 1, the specifics of the primer sequences for the nitrogen cycle functional genes are displayed.

**Table 1:** The primer sets for the amplification of functional genes involved in the nitrogen cycle.

Target gene	Primer	Function
*nifH*	*nifH* forward (GGTGGTGTMGGATTCACACARTAYGCWACAGC)	Ammonification (Rösch and Bothe, 2005)
	*nifH* reverse (TTCATTGCRTAGTTWGGRTAGT T)	
*AOA-amoA*	CrenamoA23F (ATGGTCTGGCTWAGACG)	Nitrification (Long et al., 2012)
	CrenamoA616R (GCCATCCATCTGTATGTCCA)	
*AOB-amoA*	Bac-amoA-1F (GGGGTTTCTACTGGTGGT)	Nitrification (Szukics et al., 2011)
	Bac-amoA-2R (CCCCTCKGSAAAGCCTTCTTC)	
*narG*	*NifH*F (TCGCCSATYCCGGCSATGTC)	Denitrification (Bru et al., 2007)
	*NifH*Rb (GAGTTGTACCAGTCRGCSGAYT CSG)	
*nirK*	*nirK*876 (ATYGGCGGVCAYGGCGA)	Denitrification (Barta et al., 2010)
	*nirK*1040 (GCCTCGATCAGRTTRTGGTT)	

An ABI Model 7,300 Fluorescent Quantitative PCR instrument (Applied Biosystems, United States) and ChamQ SYBR Color qPCR Master Mix (2X) reagent (Nanjing Novozymes Biotechnology Co., Ltd.) were used to perform real-time fluorescence quantitative PCR (q-PCR). The final reaction mixture volume for the quantification technique was 20 μl and contained 10 μl 2X Taq Plus Master Mix, 1 μl template DNA, 0.8 μ primer F (5 μM), 8 μl primer R (5 μM), and 7.4 μl ddH_2_O. Each qPCR reaction was run in triplicate for each set of primers, and the non-template control served as a negative control. Furthermore, q-PCR was carried out using a three-step thermal cycling method that involved 35 cycles of 95°C pre-denaturation for 5 min, 95°C denaturation for 30 s, 58°C annealing for 30 s, and 72°C extension for 1 min (Li et al., 2022). Standard curves were created by diluting pMD18-T, a plasmid for five functional genes, in a 10-fold gradient. Additionally, the *R*^2^ value of each standard curve exceeded 0.95, demonstrating a linear relationship throughout the concentration range used in this work, with amplification effectiveness ranging from 89.28 to 103.81% (mean 96.31%).

### 2.5. Data analysis

R (4.1.2), SPSS (19.0), and Excel 2019 were used for data analysis and graphing. The variations in the soil physicochemical parameters, as well as the compositional structure and diversity of the soil bacterial communities and the nitrogen cycle functional genes, in the Cr stress time series were evaluated using ANOVA. The ‘ggtern’ package in R was used to create ternary phase diagrams to illustrate the distribution variations in the phylum and genus levels of the bacterial communities in different samples (Hamilton and Ferry, 2018). Mothur (V1.30.2) was used to calculate the *α*-diversity indices (Shannon index and Simpson index) to reflect the variation in the *α*-diversity of the soil bacterial communities and the functional genes involved in the nitrogen cycle. QIIME software was used to calculate the weighted (Bray–Curtis) and unweighted (UniFrac) diversity indices in order to compare and contrast the makeup of the soil bacterial communities and functional genes involved in the nitrogen cycle in the Cr stress time series. Non-metric multidimensional scale analysis (NMDS) was used to rank and analyze the temporal distribution patterns of the soil bacterial communities and nitrogen cycle functional genes in the Cr stress time series; the results were verified by stress values, and the graphs were typically thought to have some explanatory significance when the stress<0.2. Redundancy analysis (RDA) was used to examine the relationship between the bacterial community, nitrogen cycle functional genes, and environmental factors (Oksanen et al., 2020). The ‘ggcor’ package in R was used to map the relationships between the dominant bacterial phylum, functional genes involved in the nitrogen cycle, and soil physicochemical parameters. Mantel analysis and the PLS-EM model were used to further investigate the strength of the correlations between the soil physicochemical parameters, bacterial communities, and functional genes involved in the nitrogen cycle (Li Y. et al., 2019).

## 3. Results and analysis

### 3.1. Soil physicochemical parameters in Cr-contaminated soils

**Figure 1:**
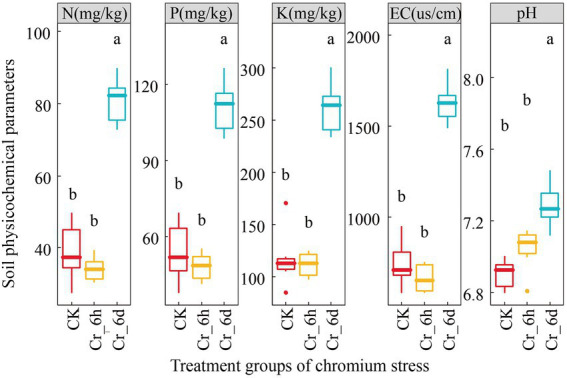
Variation trend of the soil physicochemical parameters in chromium-contaminated soils. CK, control treatment; Cr_6h, Cr stress for 6 h; Cr_6d, Cr stress for 6 days. Different letters indicate significant differences among treatments at the *p* < 0.05 level. The upper, middle, and lower lines of the box chart represent the upper quartile, median, and lower quartile, respectively, and the vertical line is the error bar.

The results of ANOVA (Figure 1) of the physicochemical parameters of the soil revealed that there was no significant difference (*p* > 0.05) between the CK and Cr_6h groups, but that there was a difference between the Cr_6h and Cr_6d groups (*p* < 0.05). At the Cr_6d stage, the soil physicochemical parameters pH and EC value, N, P, K content increased substantially by 135.79, 131.02, 135.34, and 3.55%, respectively (*p* < 0.05).

### 3.2. Growth analysis of millet seedlings in Cr-contaminated soils

In order to assess the impact of Cr stress on grain growth, measurements of the plant stem length, root length, dry weight, fresh weight, chlorophyll content, and nitrogen content were conducted (Supplementary Figure S1). ANOVA (Supplementary Table S1) revealed that stem length and chlorophyll of cereals significantly decreased by 22.40 and 19.07% (*p* < 0.05) at Cr_6h stage; stem length significantly increased by 8.67% (*p* < 0.05) at Cr_6d stage; and root length significantly decreased by 26.31% (*p* < 0.05) at Cr_6d stage compared to the CK.

### 3.3. Composition analysis of soil bacterial community and nitrogen cycle function genes in Cr-contaminated soils

#### 3.3.1. Analysis of the composition and structure of the bacterial community

A total of 791,427 high-quality sequences were discovered for the bacterial community in soil according to the high-throughput sequencing. The sparsity curve trend tended to be flat, indicating that the sequencing volume matched the analytical requirements, and 3,509 taxonomic units of bacteria were compared based on a 97% threshold for sequence similarity.

The ternary phase diagrams were plotted to represent the distribution of population types at the level of bacterial phylum (Supplementary Figure S2A) and genus (Supplementary Figure S2B) communities in soil across the time period of Cr stress. The contributions of the three groups of samples to the relative abundance of each microorganism led to the varied points in the plots or the locations of the various bacterial communities. All of the bacterial phylum and genus level communities were dispersed between 20 and 40% of the data, showing that the distribution of the bacterial phylum and genus level communities was more consistent in the Cr stress time series.

**Figure 2:**
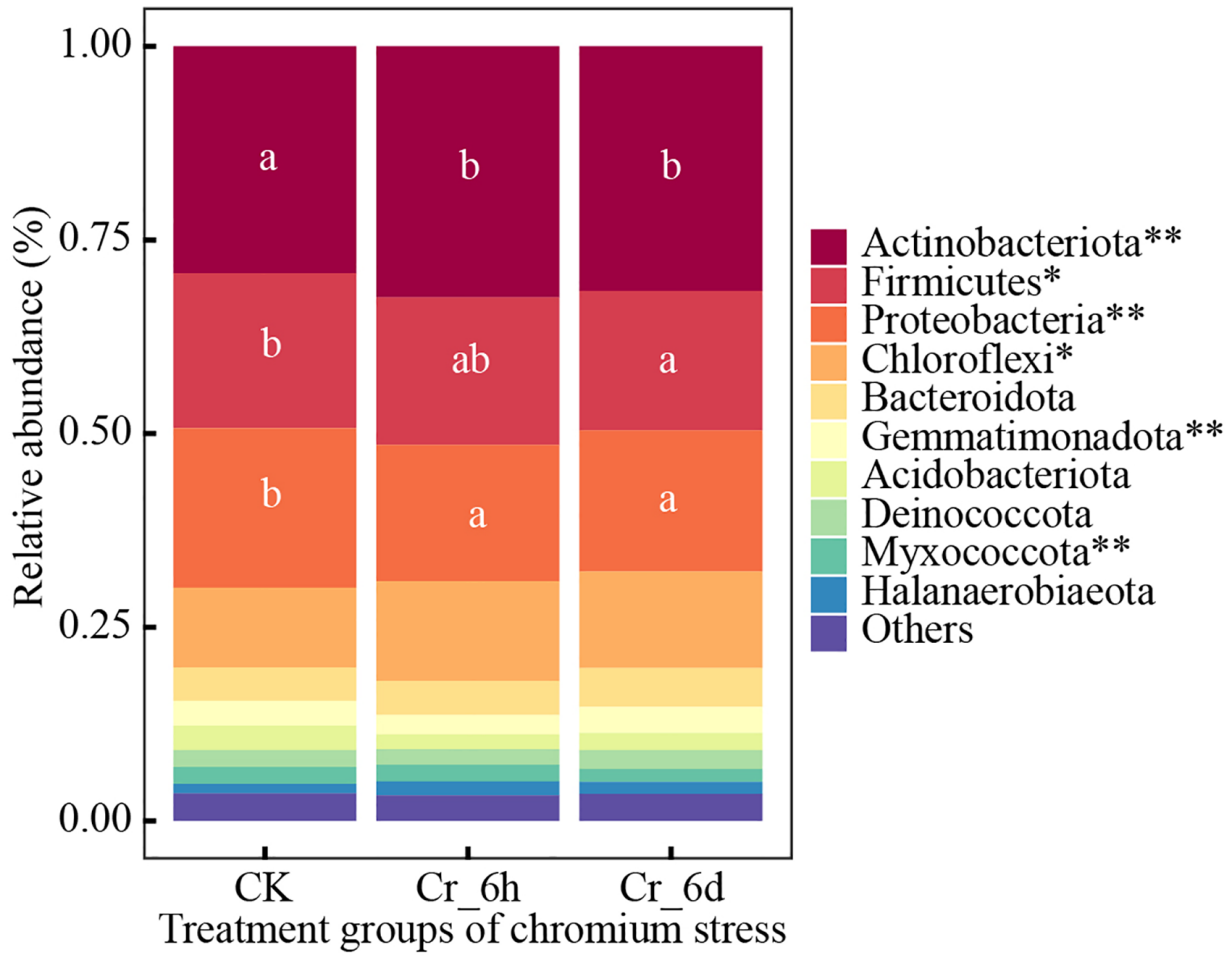
Phylum level bacterial community structures and differences among treatments in chromium-contaminated soils. Significance (P), *p ≤ 0.05, **p ≤ 0.01, ***p ≤ 0.001; CK, control treatment; Cr_6h, Cr stress for 6 h; Cr_6d, Cr stress for 6 days. Different lowercase letters indicate significant differences between components (*p* < 0.05). ‘Others’ represents the sum of the relative abundance of the remaining bacteria.

ANOVA was used to examine trends of the top 10 bacteria phylum and genus communities in soil (Supplementary Table S2), as shown in Figure 2. The top five bacterial phyla were Actinobacterota (31.11%), Firmicutes (19.00%), Proteobacteria (18.82%), Chloroflexi (11.87%), and Bacteroidota (4.57%). In the time series of Cr stress, the phylum Actinomycetes (*p* < 0.01) and Chloroflexi (*p* < 0.05) both displayed highly significant increases followed by decreases, while Firmicutes (*p* < 0.05) and Myxococcota (*p* < 0.01) displayed significant decreases; the phyla Proteobacteria (*p* < 0.01) and Gemmatimonadota (*p* < 0.01) both displayed highly significant decreases followed by increases.

According to Supplementary Figure S3 and Supplementary Table S3, the top five bacterial genera were *Planifilum* (6.34%), *Longispora* (4.59%), *Actinomadura* (2.66%), *Haloactinopolyspora* (2.51%), and *Truepera* (2.22%). In the Cr stress time series, the genus *Truepera* (*p* < 0.001) displayed a highly significant decline, whereas *Haloactinopolyspora* (*p* < 0.05) displayed a significant increase followed by a decrease.

#### 3.3.2. Analysis of variations in the abundance of genes involved in nitrogen cycle functions

**Figure 3:**
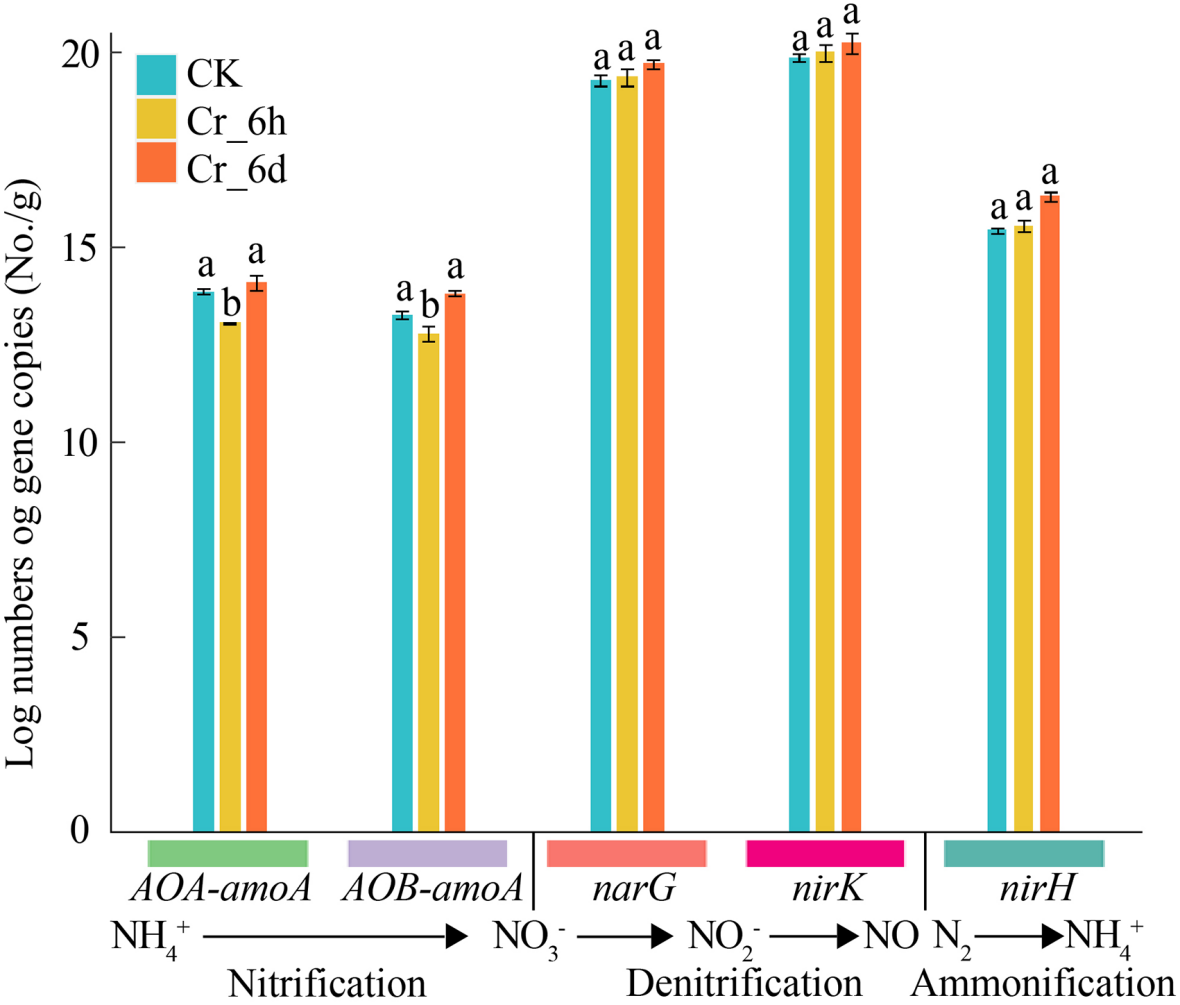
Abundance of functional genes involved in nitrogen cycling in chromium-contaminated soils. Error bars represent standard errors (*n* = 3); different lowercase letters indicate significant differences between components (*p* < 0.05).

The expression levels of the nitrogen cycle functional genes *AOA-amoA*, *AOB-amoA*, *narG*, *nirK*, and *nifH* were measured by q-PCR to clarify the impact of Cr stress on these genes (Figure 3). In the Cr stress time series, the gene abundances of *AOA-amoA* and *AOB-amoA* dramatically decreased by 5.78 and 3.75% (*p* < 0.05) at the Cr_6h stage and increased considerably by 7.89 and 8.30% (*p* < 0.05) at the Cr_6d stage. In contrast, the gene abundances of *nifH*, *narG*, and *nirK* increased at the Cr_6h stage and by 4.97, 1.84, 1.27%, respectively, at the Cr_6d stage. As a result, there was an overall increase in the expression of the nitrogen cycle–related functional genes in soil under Cr stress.

### 3.4. Diversity analysis of soil bacterial communities and nitrogen cycle functional genes in Cr-contaminated soils

#### 3.4.1. *α*-Diversity analysis

**Figure 4:**
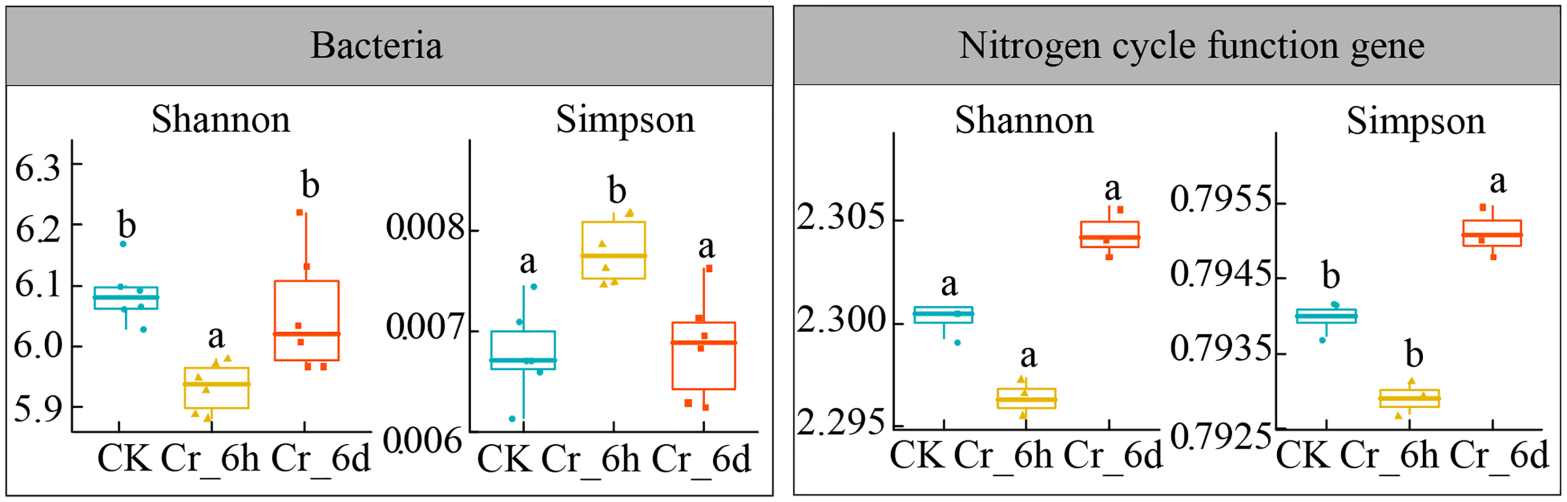
Analysis of the bacterial community *α*-diversity indexes in chromium-contaminated soils. CK, control treatment; Cr_6h, Cr stress for 6 h; Cr_6d, Cr stress for 6 days. Different letters indicate significant differences among treatments at the *p* < 0.05 level. Dithered points indicate the remaining data points, preventing points of the same value from being hidden. The upper, middle, and lower lines of the box chart represent the upper quartile, median, and lower quartile, respectively, and the vertical line is the error bar.

ANOVA was used to analyze the patterns of the bacterial community and the diversity of nitrogen cycle functional genes in agricultural soils before and after Cr stress (Shannon index, Simpson index, Figure 4). The Shannon index of the bacterial community showed a significant decrease followed by an increase (6.09, 5.93, 6.05, *p* < 0.05), while the Simpson index revealed a significant increase followed by a decrease (0.0068, 0.0078, 0.0068, *p* < 0.05). In contrast, only the Simpson index of the functional genes involved in the nitrogen cycle increased significantly at Cr_6d stage (0.794, 0.793, 0.795, *p* < 0.05). According to the results, the bacterial community displayed a phase trend of increasing and then decreasing, while the variety of nitrogen cycle functional gene diversity overall significantly decreased. As a result, the α-diversity of soil bacterial communities and nitrogen cycle functional genes in agricultural fields changed significantly before and after Cr stress.

#### 3.4.2. *β*-diversity analysis

**Figure 5:**
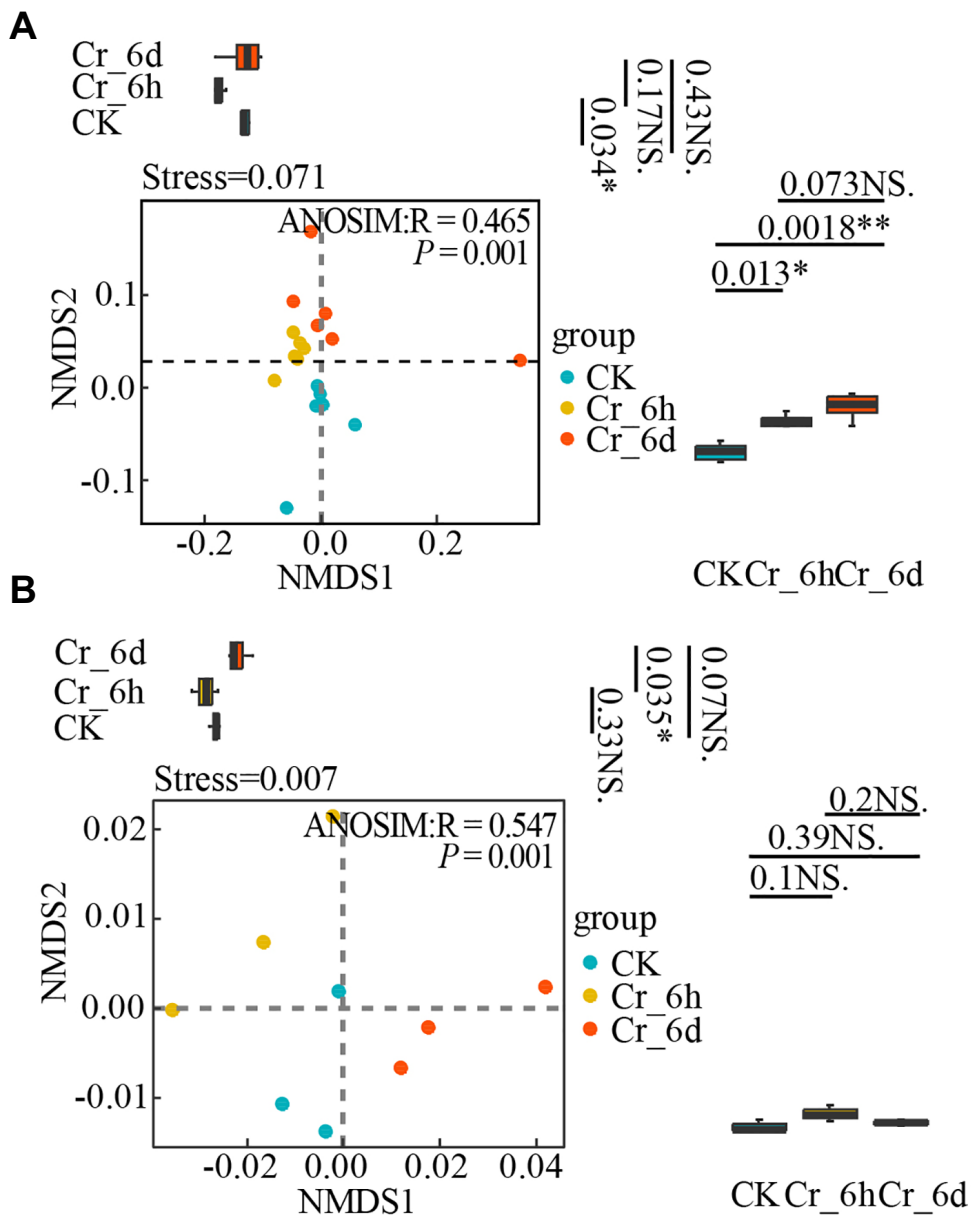
Non-metric multidimensional scale analysis of bacterial communities **(A)** and nitrogen cycle functional genes **(B)** in chromium-contaminated soils. Significance (P), *p ≤ 0.05, **p ≤ 0.01, ***p ≤ 0.001, NS, no significant difference; CK, control treatment; Cr_6h, Cr stress for 6 h; Cr_6d, Cr stress for 6 days. The *R* value indicates whether there is a difference between different groups, and *R* > 0 indicates that the difference between groups is greater than the difference within groups. Points of different colors or shapes represent samples of different treatments. The closer the two sample points, the more similar the species composition of the two samples. The upper, middle, and lower lines of the box chart represent the upper quartile, median, and lower quartile, respectively, and the vertical line is the error bar.

Based on the abundance of the bacterial phylum level community and nitrogen cycle functional gene expression in soil, non-metric multidimensional scale analysis (NMDS) and analysis of similarities (ANOSIM) were used to examine the diversity of both in the Cr stress time series (i.e., temporal distribution pattern, Figure 5). To further illustrate unique inter-group variability, grouped box plots with PC1 values as the y-axis (right side of Figure 5) and PC2 values as the y-axis (top side of Figure 5) were also presented. The results showed that the soil bacterial community structure (Figure 5A, Stress = 0.071, *p* = 0.001) and the nitrogen cycle functional genes (Figure 5B, Stress = 0.007, *p* = 0.001) were clustered within the same stage of Cr stress, but they were more distinct and spread at various stages. In Figure 5A, the box line plot on the right side demonstrates a significant difference in bacterial communities between the CK&Cr_6h and CK&Cr_6d groups (*p-*values 0.013 and 0.0018, respectively), and the box line plot on the top side demonstrates a significant difference in bacterial communities between the CK&Cr_6h groups (*p* = 0.034). In Figure 5B, only the box line plot (top side) revealed a significant difference in the functional genes between the Cr_6h and Cr_6d groups (*p* = 0.035). All of the aforementioned results show that the temporal distribution patterns of the bacterial communities and functional genes involved in the nitrogen cycle in soil changed noticeably in the Cr stress time series.

### 3.5. Association analysis of soil physicochemical parameters with bacterial communities and functional genes of the nitrogen cycle

#### 3.5.1. RDA analysis of physicochemical parameters and bacterial phylum level and functional genes

**Figure 6:**
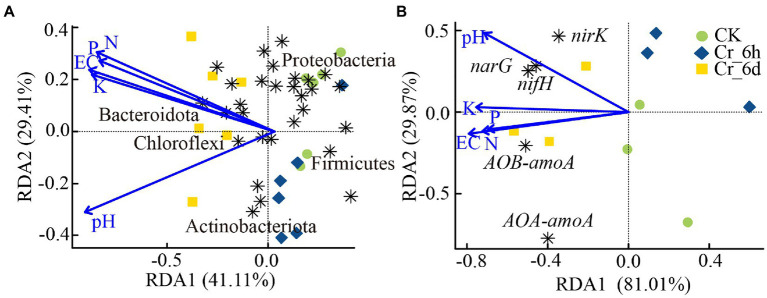
Relationship between soil physicochemical parameters and bacterial communities **(A)** and nitrogen cycle functional genes **(B)** revealed by redundancy analysis. The horizontal and vertical coordinates in the Figure indicate the degree to which the soil environmental factors explain the bacterial community structure **(A)** or phenotype **(B)**. The arrow in the Figure indicates the environmental factor, and the length of the line indicates the degree of correlation between the environmental factor and the bacterial community structure **(A)** or phenotype **(B)**. The longer the line, the greater the correlation, and vice versa. The angle between the line of the arrow and the axis indicates the correlation; the smaller the angle, the higher the correlation. An acute angle indicates a positive correlation, and an obtuse angle indicates a negative correlation.

According to the results of the RDA (PERMANOVA, *p* < 0.01) of the soil bacterial phylum level communities with physicochemical parameters, the first two RDA axes combined accounted for 70.52% of the total variation (Figure 6A). The pH of the soil had a greater impact on Chloroflexi and others, while Gemmatimonadota, Bacteroidota, and others were more impacted by the soil N, P, K, and EC concentrations. The ‘vegan’ package was also used to perform the Mantel test (Pearson correlation index) to examine the relationship between various environmental factors and changes in the bacterial community structure. The results of the test between the bacterial community and environmental factors (Supplementary Figure S3; Supplementary Table S4) were Mantel statistic *r*: 0.1967, *p* > 0.05, indicating that there was no statistically significant association between the environmental factors and the overall structural alterations of the bacterial community.

Redundancy analysis of the nitrogen cycle functional genes and soil physiochemical properties revealed (Figure 6B) that the first two RDA axes combined explained 90.88% of the overall variation. Among these properties, pH had a greater impact on *nifH*, *narG*, and *nirK*, whereas EC, N, and P had a greater impact on *AOB-amoA*. Further analysis of the relationship between various environmental factors and the abundance of the nitrogen cycle-functional genes was carried out using the Mantel test (Pearson correlation index). The results of the test between functional genes involved in the nitrogen cycle and environmental factors (Supplementary Figure S4; Supplementary Table S5) were Mantel statistic *r*: 0.4195, *p* < 0.01, indicating that the abundance of the nitrogen cycle functional genes was statistically significantly correlated with environmental factors. Additionally, the abundance of functional genes was strongly linked to N (*r* = 0.36, *p* = 0.024), K (*r* = 0.38, *p* = 0.025), and EC (*r* = 0.42, *p* = 0.01), with EC having a significant influence.

#### 3.5.2. Correlation analysis of physicochemical parameters with the dominant bacterial population and functional genes

Because all the bacterial phyla in soil were analyzed using RDA, the ggcor package was used to further map the correlation analysis. The Bray–Curtis distance was calculated for the dominant bacterial species and functional genes, and the Euclidean distance matrix of the soil physicochemical parameters was developed to clarify the Mantel association among these three, allowing an extensive investigation of how environmental factors influence functional genes and dominant bacterial communities.

**Figure 7:**
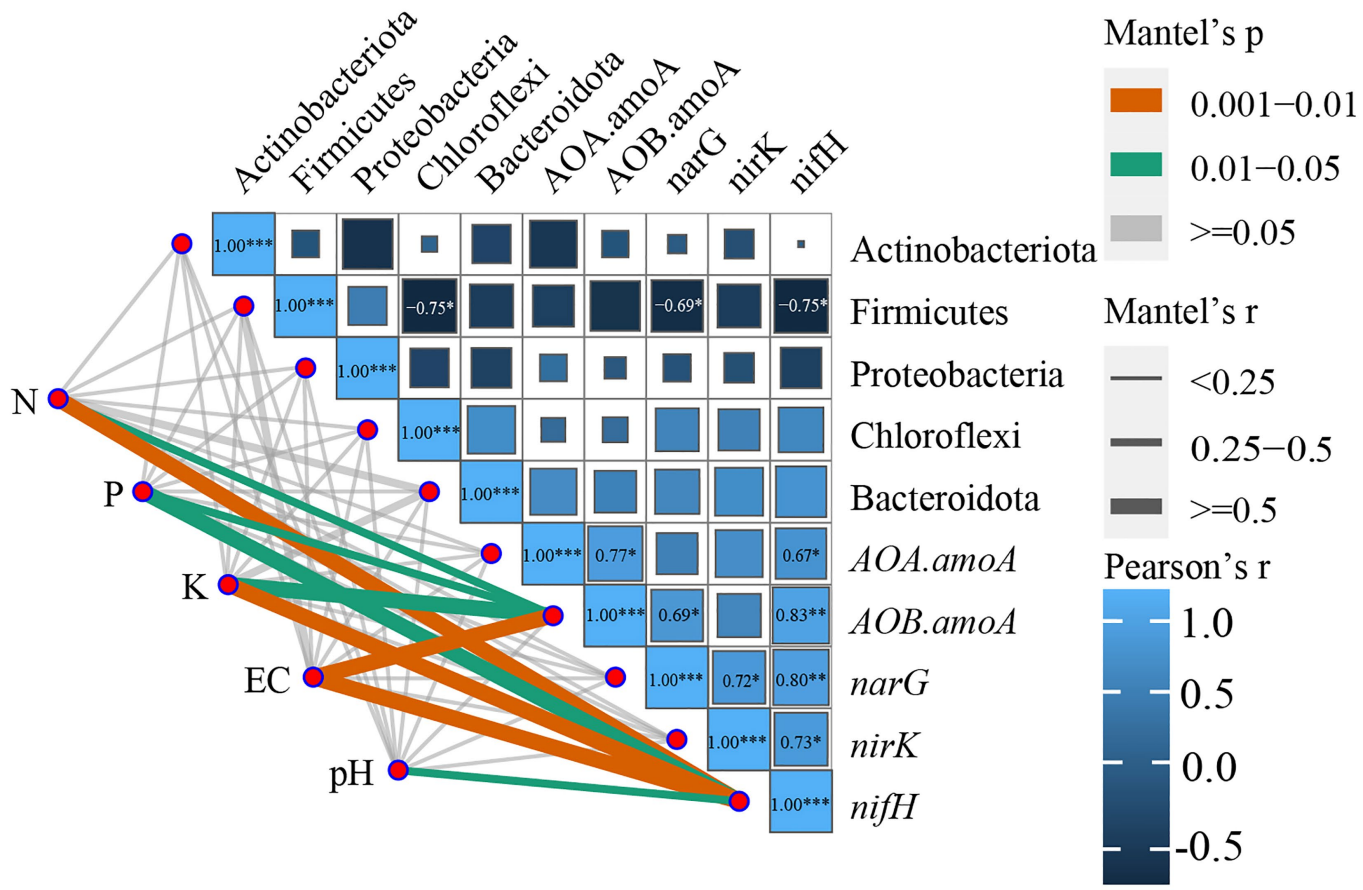
Correlation analysis of functional genes of the nitrogen cycle with environmental factors and the dominant bacterial community. *p ≤ 0.05, **p ≤ 0.01, ***p ≤ 0.001. Only significant correlations are presented as colored lines, and the color of the line corresponds to the strength of the significance of the correlation; the thickness of the line represents the magnitude of the Mantel test *r*-value.

The correlation analysis of the soil dominant bacterial community with the nitrogen cycle functional genes and soil physicochemical parameters (Figure 7; Supplementary Table S6) revealed that EC, N, and K in the soil were highly significantly correlated with *nifH* (*p-*values were 0.008, 0.006, and 0.01, respectively); pH was significantly correlated with *nifH* (*p* = 0.026, *p* < 0.05); while P and K were significantly correlated with *AOB-amoA* (*p*-values were 0.02, 0.015, respectively, *p* < 0.05). Firmicutes were substantially negatively associated (*p* < 0.05) with Chloroflexi, *narG*, and *nifH*, whereas *nifH* was significantly negatively correlated (*p* < 0.05) with *AOA-amoA* and *nirK* and extremely significantly positively correlated (*p* < 0.01) with *AOA-amoA* and *narG*; *narG* was significantly positively correlated with *nirK*. According to the aforementioned findings, there was a significant correlation between the soil physicochemical parameters, dominant bacterial phylum, and genes that function in the nitrogen cycle. However, the soil physicochemical parameters were significantly connected only with the nitrogen cycle function genes.

#### 3.5.3. Pathway effects of physicochemical parameters, dominant bacterial populations and functional genes

**Figure 8:**
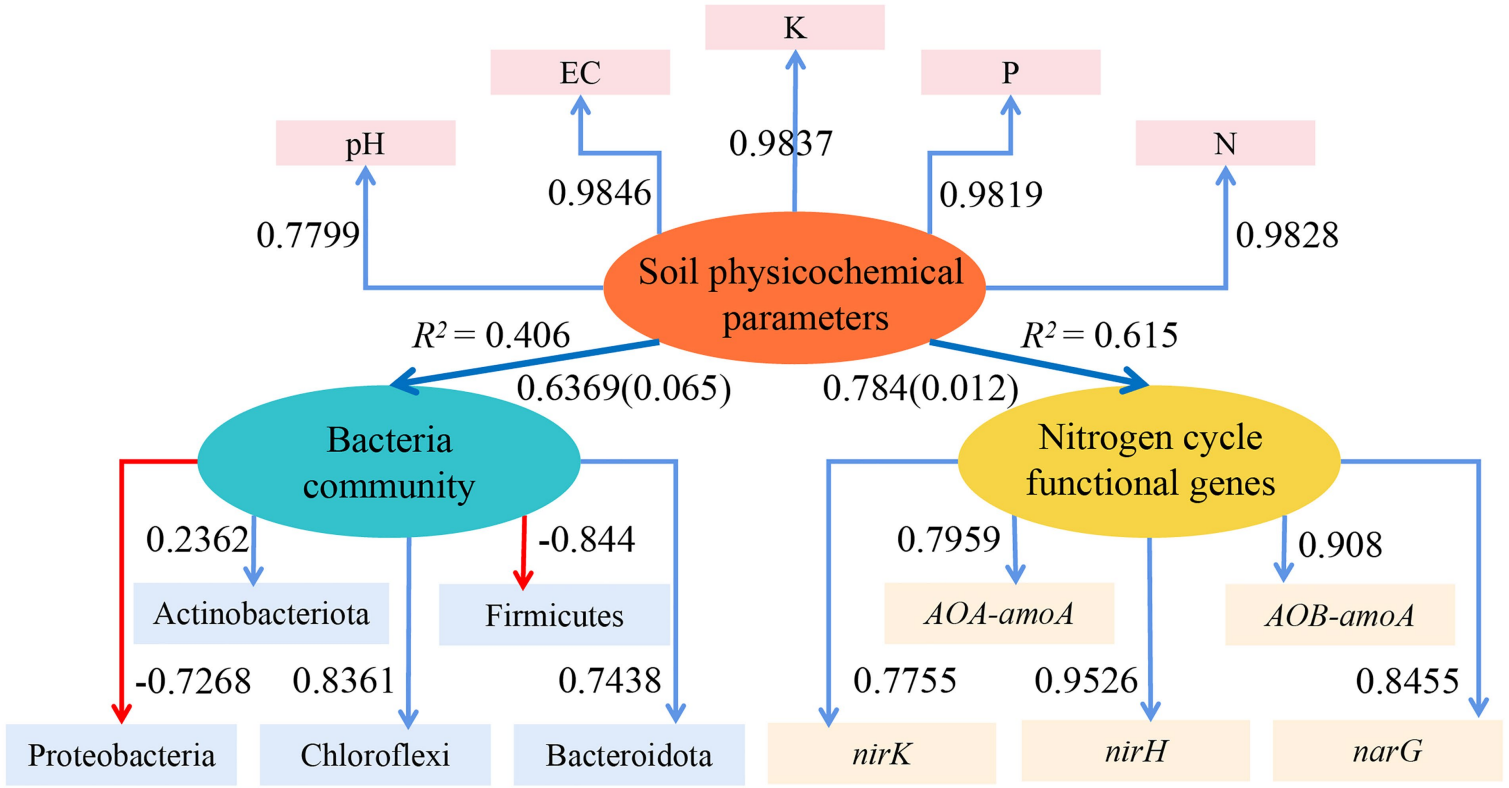
PLS-EM model to analyze the effect of the soil physicochemical parameters on the dominant bacterial community and functional genes of the nitrogen cycle. Blue arrows in the Figure indicate positive correlations, red arrows indicate negative correlations, numbers on the arrows indicate standardized path coefficients, and values in parentheses are significant *p-*values; ovals indicate potential variables, and squares are measured variables; *R*^2^ next to the variables is the explained variation.

With the use of the ‘plspm’ package in R (4.1.2), a reflective measurement model of the effects of the soil physicochemical parameters on the dominant bacterial population and functional genes involved in the nitrogen cycle was created based on the partial least squares path model (PLS-PM, Figure 8). The model had a good fit, as indicated by the goodness of fit (GOF), which was 0.6034. The dominant bacterial community and the functional genes involved in the nitrogen cycle were directly influenced by the soil physicochemical parameters (positive path and path coefficient: 0.784 and 0.6369, respectively), and the nitrogen cycle functional genes were significantly impacted (*p* = 0.012). The PLS-EM results further demonstrated that physicochemical parameters in soil had a stronger influence on functional genes than bacterial communities.

## 4. Discussion

### 4.1. Mechanisms of bacterial community structure and diversity in soils in response to Cr stress

According to the experimental findings, there were significant differences between the soil bacterial communities at the phylum and genus levels in the Cr stress time series in terms of their composition, structure, and diversity.

Similar to the findings of Zhang et al. (2021), Actinobacteriota, Firmicutes, and Proteobacteria were the three taxa with the highest abundance at the phylum level, and *Planifilum*, *Longispora*, and *Actinomadura* were the three with the highest abundance at the genus level. At the Cr_6d stage, the abundances of Actinobacteriota, Firmicutes, and Chloroflexi in the soil significantly decreased as the Cr stress time increased, whereas the abundances of Proteobacteria and Gemmatiminadota significantly increased. This may be due to the ability of soil microorganisms to adjust to various levels of heavy metal contamination by altering the structure of the microbial community (Li et al., 2017). Previous studies have demonstrated that the bacterial communities transferred across contamination levels in Cr-affected soils are predominantly Proteobacteria, Actinobacteriota, and Chloroflexi (Liu et al., 2019). Proteobacteria, the dominant phylum in soils contaminated with Cd and many other heavy metals, have considerable resistance to heavy metals (Jiang et al., 2019), and some of them have good Cr(VI) biotransformation efficiency (Garavaglia et al., 2010). Thus, Proteobacteria are frequently utilized as markers of Cr contamination, and their abundance increased significantly in the Cr_6d phase of Cr stress.

In addition, *Bacillus* in the Firmicutes and *Streptomyces* in the Actinobacteriota can reduce the toxic effect of Cr by reducing Cr(VI) (Liu et al., 2019) and have strong Cr tolerance, making them the dominant genera in Cr-stressed soils, and their abundance is more stable over time when Cr stress is present. At the stage of Cr_6d stress, however, the abundance of Actinobacteriota and Firmicutes decreased considerably, most likely because the concentration of heavy metals was higher than the community could tolerate, which inhibited growth and reduced the abundance of the community. Both Chloroflexi and Myxococcota have been demonstrated to be dominant in a variety of heavy metal-contaminated soils due to their tolerance and resistance to heavy metal stress (Berg et al., 2012; Hao et al., 2021); Chloroflexi, as low-nutrient bacteria, have an advantage in nutrient poor soils (Islam et al., 2019). Additionally, Actinobacteriota have been demonstrated to play a role in soil nutrient cycling (Narendrula-Kotha and Nkongolo, 2017), while Acidobacteriota are a dominant phylum in acidic environments or locations that are contaminated with heavy metals, using a variety of nitrogenous compounds as nitrogen sources for their growth (Eichorst et al., 2018). Therefore, as the main bacterial phyla, Acidobacteriota and Actinobacteriota may control the N cycle in Cr-stressed soils.

According to the *α*-diversity results, the Shannon and Simpson indices significantly increased and then decreased, and initially decreased and then increased, respectively, which also revealed that the diversity of bacterial communities in soil exhibited a phase change from stress to stability over the Cr stress time series. On the one hand, this might be related to the fact that the total amount of heavy metals in soils contaminated with Cd, zinc (Zn), Pb, and Cr decreases the biomass and diversity of bacterial communities and even inhibits bacterial enzymatic activities (Chen et al., 2020; Tang et al., 2022). The stage-specific changes in bacterial communities can be characterized by the extensive substrate utilization properties and high metabolic activity of some bacterial communities, which are better adapted to the toxic effects in heavy metal-contaminated soils. On the other hand, changes in α-diversity may be closely related to soil nutrients such as N, P, and SOC (Soil Organic Carbon), as soil nutrients may be the main source of energy in promoting the growth and metabolic processes of bacterial communities (Li et al., 2021). Additionally, soil microbes are crucial for material and nutrient cycling in plant and soil ecosystems, but the mechanisms driving the composition and diversity of microbial communities vary depending on the kind of vegetation. According to research (Xu et al., 2021), vegetation traits and soil physicochemical features may synergistically regulate microbial communities in soils. Furthermore, because heavy metals have more complex natural origins and soil biogeochemical characteristics and result in complex interactions between microbial communities, multiple factors combine to determine whether an ecosystem stimulated by metals would produce a positive or negative response (Abdu et al., 2017). In summary, bacterial communities in soils adapt to Cr contamination through phase changes in composition, structure, and diversity; rather than solely being influenced by the toxic effects of heavy metals, these phase shift characteristics of bacterial community diversity in soil may be influenced by a number of factors, including heavy metal contamination, soil nutrients, and vegetation conditions.

### 4.2. Mechanisms of nitrogen cycle functional gene abundance and diversity in soil in response to Cr stress

According to the experimental data, there were significant differences in the composition structure and diversity of functional genes involved in the nitrogen cycle in soil under conditions of Cr stress.

The *AOA-amoA* and *AOB-amoA* abundances exhibited a significant decrease and then an increase (*p* < 0.05). In particular, the abundance of the *AOB-amoA* gene increased by 8.30% in the Cr_6d stage compared with the Cr_6h stage; the abundances of *nifH*, *narG*, and *nirK* showed an increasing tendency; and the gene abundance was ranked as denitrification > nitrogen fixation > nitrification. The results of α-diversity analysis revealed that from Cr_6h to Cr_6d, the diversity of the nitrogen cycle functional genes significantly decreased. Similar to the bacterial community diversity, the functional gene abundance displayed a stress to stability phase transition. This is most likely because high Cr pollution temporarily inhibits the nitrogen cycle function in soil, which is caused by the soil microbial community’s immediate damage response to external stimuli and the gradual recovery of the nitrogen cycle function with increasing Cr stress time (Zhou et al., 2016). It has been demonstrated that Cd stress promotes N cycling activities in soil and that the degree of stress, microbial response mechanisms, and other mechanisms change with time (Zhao et al., 2021), which is consistent with the present study’s findings. The initial and rate-limiting phase of soil nitrification is the ammonia oxidation process, which is mediated by AOA and AOB genes with significant changes in abundance. As specialized chemoautotrophic bacteria, AOA and AOB can oxidize ammonia to nitrite and generate huge amounts of ATP, which promotes the survival of microbial communities in soil (Prosser et al., 2020). Similar to other research, the results showed that AOA was more abundant than AOB, probably because AOA is the dominating microbe in soil ammonia oxidation (Leininger et al., 2006) and is better adapted to the challenging environment of low oxygen and oligotrophic growth (David et al., 2014). Although AOA and AOB populations exhibit ecological niche differentiation and functional complementarity (Prosser and Nicol, 2012), AOB populations are more vulnerable to Cr stress than AOA, and therefore their abundance changes more significantly.

In the Cr stress time series, the abundance of the nitrogen cycle functional genes generally increased. This may be explained by the fact that soil microbial communities in Cr-stressed environments protect themselves from metal toxicity using a variety of resistance mechanisms, such as intercellular isolation and enzymatic detoxification, while dominant phyla such as Actinomycetes and Acidobacteria may enhance the transcriptional processes of genes related to amino acid biosynthesis and uptake pathways for protein detoxification (Viti et al., 2014). Thus, as the succession process advances, the soil microbial community gradually adjusts to external pressures at the physiological, genotypic, or community level (Spang et al., 2012; Azarbad et al., 2015) and inevitably requires more energy and irreversible overall metabolic transformation in order to maintain higher energy expenditures in organism growth and metabolic activities (Singh et al., 2014); thus, the nitrogen use and transformation process may be expedited. However, during the Cr_6h phase, Cr stress inhibited bacterial communities and nitrogen cycle functional gene abundance in soil, probably because most denitrifying bacteria, diazotrophs, and soil bacteria are mostly heterotrophic and primarily take up ammonia to biosynthesize compounds required for metal detoxification. Based on the energy saving strategy of the soil microbial community, in order to save energy costs, the soil microbial community may inhibit processes that require high energy, such as cell division (Chen et al., 2016), so its abundance tends to decrease. In conclusion, Cr stress may accelerate the process of N utilization and transformation in soil, and soil microbial communities may adopt energy production and conservation strategies to survive in Cr-stressed environments.

### 4.3. Association of soil physicochemical parameters with bacterial communities and nitrogen cycle functional genes

The findings revealed that at the Cr_6d stage, all soil physicochemical parameters increased significantly (*p* < 0.05); pH had a greater impact on Chloroflexi, while the N, P, K, and EC contents primarily affected Gemmatimonadota and Bacteroidota, and the functional genes involved in the nitrogen cycle *nifH* and *AOB-amoA* were significantly correlated with EC, N, and K, with EC having the strongest effect. Similar to the findings of Xu et al. (2019), pH had an impact on the bacterial community and was significantly correlated with *nifH* functional genes. First, it is possible that pH directly imposes a certain degree of physiological restriction on soil microorganisms; its value outside of a certain range will change how microorganisms interact, which will have an impact on the abundance of microbial taxa that cannot grow individually (Lauber et al., 2009). Second, previous research has demonstrated that soil pH affects the transport and bioavailability of heavy metals (Liang et al., 2017), and in addition to influencing the composition of the microbial community, it also regulates the diversity and abundance of functional genes involved in the nitrogen cycle (Lauber et al., 2009; Rousk et al., 2010). Additionally, Kandeler et al. (2006) demonstrated that pH had little to no impact on the functional genes involved in denitrification, which is in line with the findings of the present study. A wide-ranging variable in soil, pH is strongly related to properties such as the moisture content and nutrient availability. By modifying soil conditions, pH may cause variations in microbial composition; consequently, other soil physicochemical parameters other than soil pH may also have a limiting impact on the spread of microbial communities. For example, it has been demonstrated that the microbial community and available potassium in soil are significantly and negatively correlated (Pan et al., 2020), which may result from the enrichment of microorganisms that prefer potassium, such as *Paenibacillus* that dominate the soil, thus reducing microbial diversity.

The nitrification process of soil bacteria is favored by an elevated N content in the soil (Ouyang et al., 2017); hence, AOB is strongly associated with N when the N concentration increases. One study demonstrated that AOB are susceptible to environmental factors, and high pH and EC directly affect the variety of AOB in soil (Wang and Gu, 2014). In contrast, AOA require less energy and ammonium than AOB, and they have mechanisms for adapting to pH and severe settings, making AOA better suited for surviving in soil environments that are stressed by heavy metals. In Cr-contaminated soils, the soil may constitute a boundary habitat affected by heavy metals at the initial stage of stress, when nutrients are scarce and the concentration of effective carbon and nitrogen is low, thus the colonization of nitrogen-fixing bacteria enhances the input of nitrogen into the soil (Schrama et al., 2012). Furthermore, the *nifH* genes in this study had a high initial abundance and gradually increased, indicating that the input of heterotrophic nitrogen may be crucial in the time series.

## 5. Conclusion

1. In Cr-contaminated soils, the bacterial community’s composition and structure at the phylum and genus levels changed significantly; the diversity of the community displayed a phase change characteristic from stress to stability. Resistant bacterial phyla such as Proteobacteria and Chloroflexi were enriched to maintain community homeostasis and adapted to Cr stress by changes in compositional structure and diversity. Their abundance and diversity alterations may be jointly influenced by heavy metals, soil physiochemical properties, and vegetation factors.
2. In Cr-contaminated soils, the quantity of the nitrogen cycle functional genes was ranked as denitrification > nitrogen fixation > nitrification, and the overall abundance typically increased while the diversity noticeably decreased. Cr stress had a considerable impact on the abundance of the *AOA-amoA* and *AOB-amoA* genes in the nitrification process, and the abundance of both genes was characterized by a transition from stress to stability. Based on the energy production and conservation strategies of microbial communities, Cr stress may have sped up the transformation and use of nitrogen in soil while reducing the abundance of the microbial communities.
3. The correlation analysis with soil physicochemical parameters, including RDA, ggcor correlation analysis, and PLS-PM, revealed that environmental factors had a greater impact on functional genes involved in nitrogen cycling than bacterial communities. Additionally, the high abundance of *nifH* and strong connection with the soil physicochemical parameters suggested that the colonization of nitrogen-fixing bacteria during the Cr stress time series may have facilitated nitrogen fixation in the environment to increase soil nutrients and facilitate the survival of the microbial community in the soil.

## Data availability statement

The original contributions presented in the study are publicly available. This data can be found here: National Center for Biotechnology Information (NCBI), accession no. PRJNA930506.

## Author contributions

XB, PZ, XZ, and XJ designed this study. XB and YL participated in the operation process of the experiment and carried out data processing. XB performed the statistical analysis and wrote the manuscript. All authors contributed to the article and approved the submitted version.

## Funding

This study was supported by the Science and Technology Innovation Programs of Higher Education Institutions in Shanxi (2020L0535), and the Construction of Innovation Discipline Cluster Servicing Valley Ecological Governance Industry (Shanxi “1331 Project”). This research was also supported by the Department of Biological Sciences and Technology.

## Conflict of interest

The authors declare that the research was conducted in the absence of any commercial or financial relationships that could be construed as a potential conflict of interest.

## Publisher’s note

All claims expressed in this article are solely those of the authors and do not necessarily represent those of their affiliated organizations, or those of the publisher, the editors and the reviewers. Any product that may be evaluated in this article, or claim that may be made by its manufacturer, is not guaranteed or endorsed by the publisher.

## Footnotes

^3^ http://drive5.com/uparse/

^4^ http://sourceforge.net/projects/rdp-classifier/

^5^ http://qiime.org/scripts/assign_taxonomy.html

^6^ https://www.mothur.org/wiki/Download_mothur
