# Supplementary material for: Response mechanisms of bacterial communities and nitrogen cycle functional genes in millet rhizosphere soil to chromium stress
Source: Front Microbiol. 2023 Feb 22;14:1116535. doi: 10.3389/fmicb.2023.1116535 (PMC9992798; doi:10.3389/fmicb.2023.1116535)
Supplement: Supplementary file 1 [file Data_Sheet_1.docx]

Supplementary Material

# Supplementary Figures and Tables

## Supplementary Figures


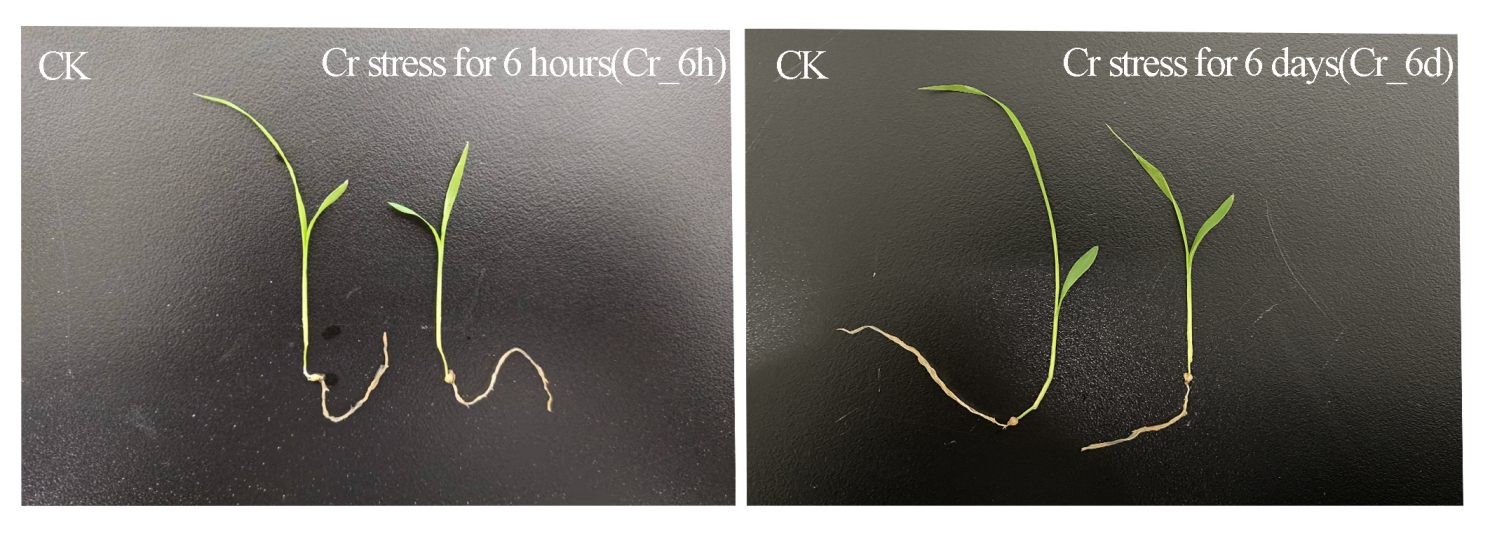


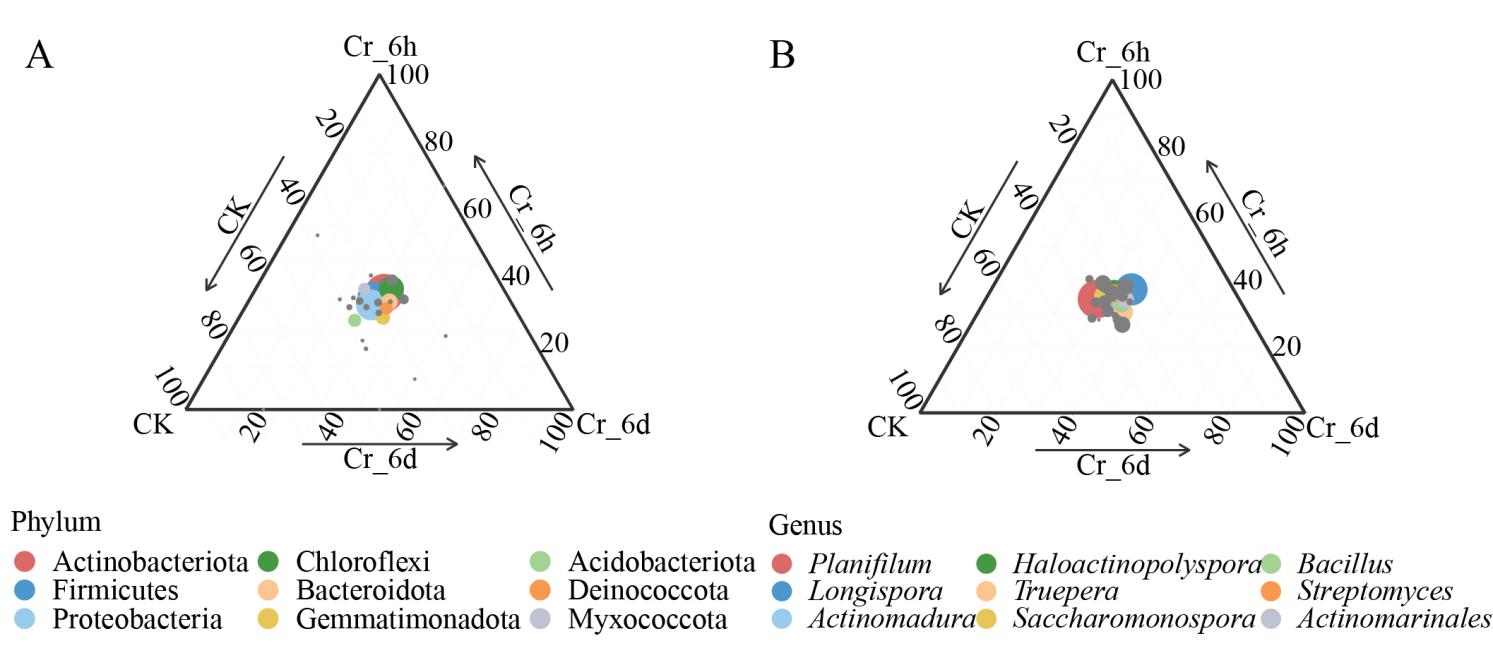
**Supplementary Figure 1.** Morphological changes of cereal seedlings after Cr stress

**Supplementary Figure 2.** Ternary phase diagram of bacterial phylum (A) and genus (B) levels in the Cr stress time series

Note: CK: control treatment; Cr_6 h: Cr stress for 6 hours; Cr_6 d: Cr stress for 6 days; the colors of the different dots in the graph represent different bacteria at the bacterial phylum and genus levels, and the sizes of the dots represent their abundance. Centrally located communities were more evenly distributed among the three sample groups, while the closer the proximity to a vertex, the more specific the enrichment of that community in that group of samples.


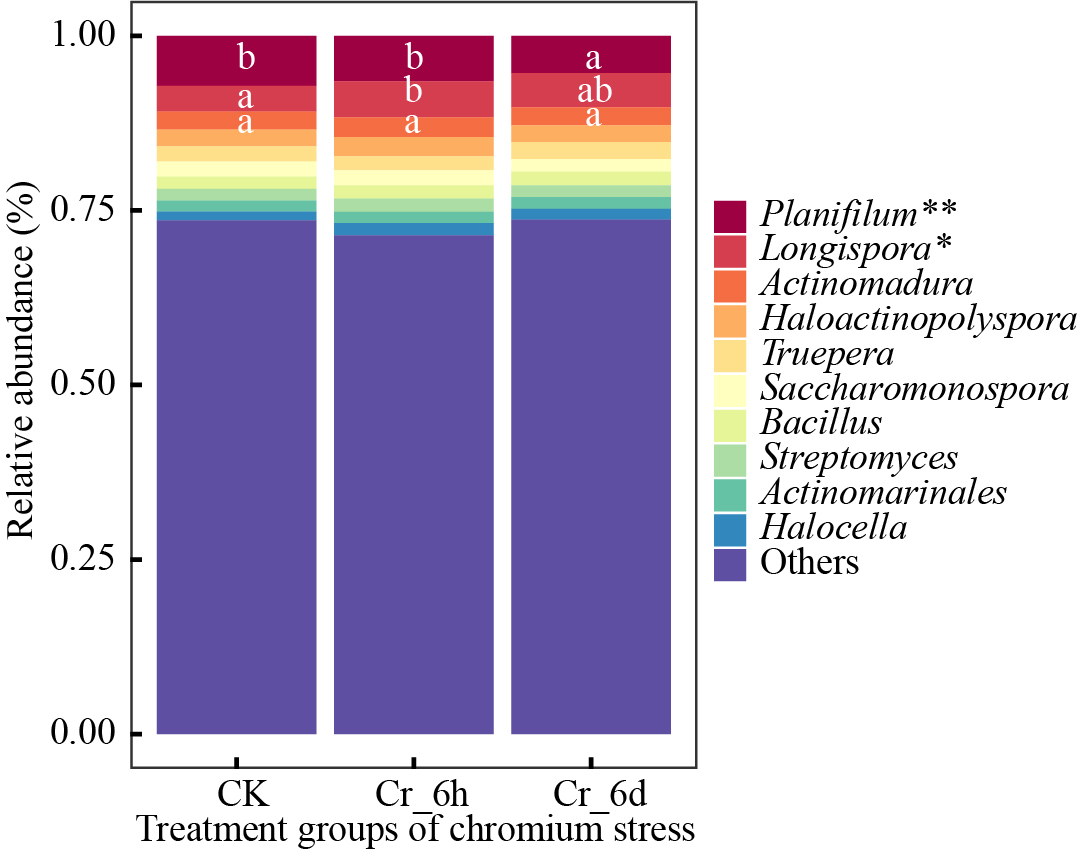
**Supplementary Figure 3.** Genus level bacterial community structures and differences among treatments in the Cr stress

Note: Significance(P), * *P*≤0.05, ** *P*≤0.01, *** *P*≤0.001; CK: control treatment; Cr_6 h: Cr stress for 6 hours; Cr_6 d: Cr stress for 6 days. Different lowercase letters indicate significant differences between components (*P*<0.05). ‘Others’ represents the sum of the relative abundance of the remaining bacteria.


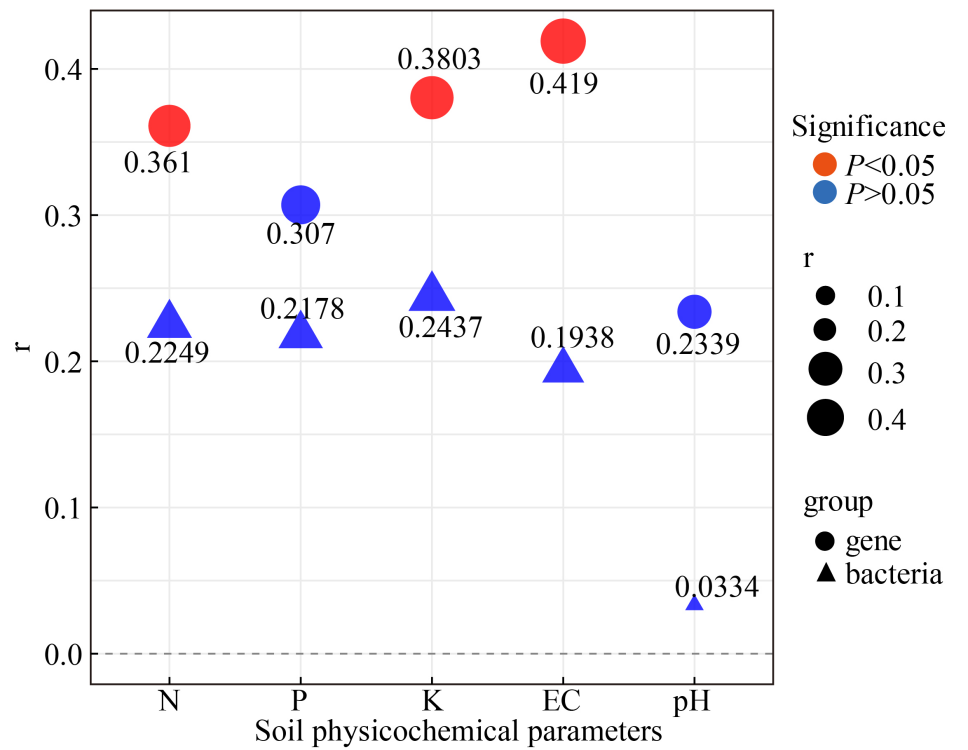


**Supplementary Figure 4.** Mantel test bubble chart

Note: The horizontal coordinates are environmental factors and the vertical coordinates are r values, with shapes to distinguish genes from species and colors to distinguish significance. Values are labeled as r in the figure and the size of the area of the bubble is set with the r value, with r=0 at the dashed line. r: Correlation between the β-diversity distance matrix of microbial communities or functional genes and the distance matrix of environmental factors. * Significance(P), *P*<0.05 indicates that the correlation between environmental factors and microbial community or functional gene distance matrix is statistically significant.

## Supplementary Tables

**Supplementary Table 1.** Millet growth and biomass determination uhder the chromium stress (mean±SE)

| Treatment group | Control group (CK) | | | Chromium stress for 6 hours (Cr_6h) | Chromium stress for 6 days (Cr_6d) |
| --- | --- | --- | --- | --- | --- |
|  | 0h | 6h | 6d |  |  |
| Stem length (cm) | 5.86±0.55e | 7.12±0.17cd | 9.37±0.50b | 5.52 ± 0.55b | 7.73 ± 1.12a |
| Root length (cm) | 4.18±0.81ab | 4.22±0.78ab | 5.19±0.57a | 3.64 ± 0.23a | 3.83 ± 0.75a |
| Dry weight (g) | 0.0033±0.0005c | 0.0043±0.0005bc | 0.0053±0.0005b | 0.0034 ± 0.0005b | 0.005 ± 0.0006a |
| Fresh weight (g) | 0.0280±0.0053de | 0.0362±0.0029cd | 0.0515±0.0031b | 0.0294 ± 0.0026b | 0.0378 ± 0.0051a |
| Chlorophyll (SPAD) | 18.54±3.89bc | 23.28±1.69a | 20.68±2.84ab | 18.84 ± 1.77b | 16.34 ± 2.00b |
| N (mg/g) | 5.27±1.94c | 7.49±0.51ab | 6.76±0.84abc | 6.19 ± 0.52b | 5.45 ± 0.58b |

Data are mean ± standard deviation, and different lowercase letters indicate significant differences between components (*P*<0.05)

**Supplementary Table 2.** Changes in abundance of bacterial phyla in chromium-contaminated soils

| Phylum | Control group (CK) | Chromium stress for 6 hours (Cr_6h) | Chromium stress for 6 days (Cr_6d) |
| --- | --- | --- | --- |
| Actinobacteriota | 0.293257462a | 0.324121394b | 0.315954477b |
| Firmicutes | 0.19959917b | 0.1905447ab | 0.179865436a |
| Proteobacteria | 0.2062415b | 0.176100494a | 0.182277575a |
| Chloroflexi | 0.102898862a | 0.128559158b | 0.124765586ab |
| Bacteroidota | 0.043117887a | 0.044069859a | 0.05003221a |
| Gemmatimonadota | 0.032037793b | 0.02481569a | 0.033641114b |
| Acidobacteriota | 0.031515282a | 0.019440269a | 0.022131558a |
| Deinococcota | 0.022052824a | 0.020098776a | 0.024407702a |
| Myxococcota | 0.021494524b | 0.021530313b | 0.016813399a |
| Halanaerobiaeota | 0.01257605a | 0.017758213a | 0.01557512a |
| Patescibacteria | 0.008710901a | 0.010500322ab | 0.012726362b |
| Planctomycetota | 0.00556152a | 0.005167848a | 0.005446997a |
| Bdellovibrionota | 0.005819197b | 0.004838594a | 0.004287453a |
| Cyanobacteria | 0.003020543a | 0.002433612a | 0.002984754a |
| Dependentiae | 0.00253382b | 0.002032782a | 0.002082886ab |
| Verrucomicrobiota | 0.002540978a | 0.001825209a | 0.001610479a |
| unclassified_k__norank_d__Bacteria | 0.002297616b | 0.001932575ab | 0.001689213a |
| Desulfobacterota | 0.000637034a | 0.000658507a | 0.000751557a |
| SAR324_cladeMarine_group_B | 0.000622718ab | 0.000858922b | 0.000386515a |
| Sumerlaeota | 0.000637034a | 0.000529669a | 0.000422303a |
| Nitrospirota | 0.000543984b | 0.000221888a | 0.000458092ab |
| MBNT15 | 0.000343569ab | 0.000465249b | 0.000236204a |
| WS2 | 0.000529669b | 0.000271992ab | 0.000193257a |
| Dadabacteria | 0.000229046a | 0.000286307a | 0.000236204a |
| Hydrogenedentes | 0.000207573a | 0.000257677a | 0.000178942a |
| WPS-2 | 0.000257677a | 0.000114523 | 0.0001861a |
| Armatimonadota | 0.000229046a | 0.000171784a | 0.000121681a |
| NB1-j | 0.000128838a | 0.000128838a | 0.000150311a |
| Fibrobacterota | 6.44192E-05a | 6.44192E-05a | 0.000164627b |
| Methylomirabilota | 0.000107365a | 5.01038E-05a | 8.58922E-05a |
| Latescibacterota | 7.15768E-05ab | 9.30499E-05b | 1.43154E-05a |
| RCP2-54 | 4.29461E-05a | 2.14731E-05a | 2.86307E-05a |
| Abditibacteriota | 2.86307E-05a | 7.15768E-06a | 4.29461E-05a |
| TX1A-33 | 7.15768E-06a | 1.43154E-05a | 2.14731E-05a |
| Entotheonellaeota | 2.86307E-05a | 0a | 7.15768E-06a |
| Fusobacteriota | 0a | 1.43154E-05a | 7.15768E-06a |
| Elusimicrobiota | 7.15768E-06a | 0a | 7.15768E-06a |

Different lowercase letters indicate significant differences between components (*P*<0.05)

**Supplementary Table 3.** Changes in abundance of bacterial genus in chromium-contaminated soils

| Genus | Control group (CK) | Chromium stress for 6 hours (Cr_6h) | Chromium stress for 6 days (Cr_6d) |
| --- | --- | --- | --- |
| *Planifilum* | 0.071541049b | 0.065170711b | 0.053503686a |
| *Longispora* | 0.036947964a | 0.051578269b | 0.049051607ab |
| *Actinomadura* | 0.025767662a | 0.028280009a | 0.025681769a |
| *Haloactinopolyspora* | 0.023978241a | 0.02747119a | 0.023920979a |
| *Truepera* | 0.022024193a | 0.020091618a | 0.024386229a |
| *Saccharomonospora* | 0.021351371a | 0.02172357a | 0.017994417a |
| *Bacillus* | 0.017715267a | 0.018753131a | 0.019461742a |
| *Streptomyces* | 0.016763295a | 0.019017966a | 0.01684203a |
| *Actinomarinales* | 0.01557512a | 0.016026054a | 0.016942237a |
| *Halocella* | 0.012568893a | 0.017758213a | 0.015567962a |

**Supplementary Table 4.** Results of mantel test for species abundance and individual environmental factors

| Factor | r | p.value | p.adjusted |
| --- | --- | --- | --- |
| N | 0.22489706 | 0.115 | 0.17125 |
| P | 0.21775503 | 0.11 | 0.17125 |
| K | 0.24372638 | 0.113 | 0.17125 |
| EC | 0.19381435 | 0.137 | 0.17125 |
| PH | 0.03340337 | 0.412 | 0.412 |

**Supplementary Table 5.** Results of Mantel test for functional gene abundance versus individual environmental factors

| Factor | r | p.value | p.adjusted |
| --- | --- | --- | --- |
| N | 0.3610459 | 0.024 | 0.04166667 |
| P | 0.3070274 | 0.041 | 0.05125 |
| K | 0.3803229 | 0.025 | 0.04166667 |
| EC | 0.4190217 | 0.01 | 0.04166667 |
| PH | 0.233878 | 0.087 | 0.087 |

**Supplementary Table 6.** Results of Mantel test of environmental factors and soil bacterial dominant flora and nitrogen cycle functional genes

| **ENV** | **SPE/GENE** | **r** | **p.value** | **r_value** | **p_value** |
| --- | --- | --- | --- | --- | --- |
| N | Actinobacteriota | -0.008694351 | 0.477 | <0.25 | >=0.05 |
| N | Firmicutes | 0.210532762 | 0.091 | <0.25 | >=0.05 |
| N | Proteobacteria | 0.048923533 | 0.291 | <0.25 | >=0.05 |
| N | Chloroflexi | -0.028102555 | 0.454 | <0.25 | >=0.05 |
| N | Bacteroidota | 0.25824654 | 0.064 | 0.25-0.5 | >=0.05 |
| N | AOA.amoA | 0.173907841 | 0.161 | <0.25 | >=0.05 |
| N | AOB.amoA | 0.486187553 | 0.027 | 0.25-0.5 | 0.01-0.05 |
| N | narG | 0.04995567 | 0.303 | <0.25 | >=0.05 |
| N | nirK | -0.005738052 | 0.471 | <0.25 | >=0.05 |
| N | nifH | 0.653638816 | 0.008 | >=0.5 | 0.001-0.01 |
| P | Actinobacteriota | 0.000918511 | 0.424 | <0.25 | >=0.05 |
| P | Firmicutes | 0.17626713 | 0.126 | <0.25 | >=0.05 |
| P | Proteobacteria | 0.094002923 | 0.202 | <0.25 | >=0.05 |
| P | Chloroflexi | -0.049129742 | 0.523 | <0.25 | >=0.05 |
| P | Bacteroidota | 0.245002262 | 0.074 | <0.25 | >=0.05 |
| P | AOA.amoA | 0.128626833 | 0.217 | <0.25 | >=0.05 |
| P | AOB.amoA | 0.448136561 | 0.02 | 0.25-0.5 | 0.01-0.05 |
| P | narG | 0.017607952 | 0.379 | <0.25 | >=0.05 |
| P | nirK | -0.048144909 | 0.554 | <0.25 | >=0.05 |
| P | nifH | 0.611333206 | 0.011 | >=0.5 | 0.01-0.05 |
| K | Actinobacteriota | -0.017961855 | 0.452 | <0.25 | >=0.05 |
| K | Firmicutes | 0.22765007 | 0.094 | <0.25 | >=0.05 |
| K | Proteobacteria | 0.140320018 | 0.152 | <0.25 | >=0.05 |
| K | Chloroflexi | 0.037645737 | 0.283 | <0.25 | >=0.05 |
| K | Bacteroidota | 0.271296895 | 0.078 | 0.25-0.5 | >=0.05 |
| K | AOA.amoA | 0.164311782 | 0.21 | <0.25 | >=0.05 |
| K | AOB.amoA | 0.546176566 | 0.015 | >=0.5 | 0.01-0.05 |
| K | narG | 0.131958922 | 0.182 | <0.25 | >=0.05 |
| K | nirK | 0.013733936 | 0.43 | <0.25 | >=0.05 |
| K | nifH | 0.734435344 | 0.006 | >=0.5 | 0.001-0.01 |
| EC | Actinobacteriota | 0.01419398 | 0.382 | <0.25 | >=0.05 |
| EC | Firmicutes | 0.255215836 | 0.052 | 0.25-0.5 | >=0.05 |
| EC | Proteobacteria | -0.003169656 | 0.361 | <0.25 | >=0.05 |
| EC | Chloroflexi | -0.077658146 | 0.588 | <0.25 | >=0.05 |
| EC | Bacteroidota | 0.203618533 | 0.11 | <0.25 | >=0.05 |
| EC | AOA.amoA | 0.172729296 | 0.154 | <0.25 | >=0.05 |
| EC | AOB.amoA | 0.567399802 | 0.007 | >=0.5 | 0.001-0.01 |
| EC | narG | 0.140446709 | 0.17 | <0.25 | >=0.05 |
| EC | nirK | 0.017679608 | 0.375 | <0.25 | >=0.05 |
| EC | nifH | 0.683229208 | 0.004 | >=0.5 | 0.001-0.01 |
| PH | Actinobacteriota | 0.02795378 | 0.375 | <0.25 | >=0.05 |
| PH | Firmicutes | 0.114818604 | 0.249 | <0.25 | >=0.05 |
| PH | Proteobacteria | 0.163315924 | 0.138 | <0.25 | >=0.05 |
| PH | Chloroflexi | 0.006124352 | 0.412 | <0.25 | >=0.05 |
| PH | Bacteroidota | -0.040566239 | 0.598 | <0.25 | >=0.05 |
| PH | AOA.amoA | -0.06687461 | 0.625 | <0.25 | >=0.05 |
| PH | AOB.amoA | 0.165535198 | 0.167 | <0.25 | >=0.05 |
| PH | narG | 0.17952264 | 0.128 | <0.25 | >=0.05 |
| PH | nirK | 0.092997164 | 0.333 | <0.25 | >=0.05 |
| PH | nifH | 0.378399144 | 0.026 | 0.25-0.5 | 0.01-0.05 |
